# Upfront Oxaliplatin–Fluoropyrimidine Chemotherapy and Somatostatin Analogues in Advanced Well-Differentiated Gastro-Entero-Pancreatic Neuroendocrine Tumors

**DOI:** 10.3390/cancers17091561

**Published:** 2025-05-03

**Authors:** Maria Grazia Maratta, Ileana Sparagna, Denis Occhipinti, Luigi Roca, Margherita Sgambato, Salvatore Raia, Antonio Bianchi, Sabrina Chiloiro, Ernesto Rossi, Guido Rindi, Giampaolo Tortora, Giovanni Schinzari

**Affiliations:** 1 Medical Oncology Unit, Comprehensive Cancer Center, Fondazione Policlinico Universitario Agostino Gemelli—IRCCS, 00168 Rome, Italy; 2 Faculty of Medicine and Surgery, Università Cattolica del Sacro Cuore, 00168 Rome, Italy; 3 Division of Endocrinology and Metabolism, Fondazione Policlinico Universitario Agostino Gemelli—IRCCS, 00168 Rome, Italy; 4 Anatomic Pathology Unit, Fondazione Policlinico Universitario A. Gemelli—IRCCS, 00168 Rome, Italy

**Keywords:** neuroendocrine tumor, gastroenteropancreatic NET, chemotherapy

## Abstract

(1) Background: GEP-NETs are frequently diagnosed at advanced stage. For well-differentiated somatostatin receptor-positive (SSTR+) NETs, SSA are the preferred first-line therapy. However, in newly diagnosed patients with G2/G3 and a high tumor burden, SSA alone might not be enough; (2) Methods: We conducted a retrospective analysis to assess the effectiveness of combining oxaliplatin–fluoropyrimidine chemotherapy with SSA as an upfront strategy in newly diagnosed metastatic G2/G3 GEP-NET patients treated with oxaliplatin–fluoropyrimidine-based chemotherapy; (3) Results: Between March 2017 and October 2023, 32 pts (19 males, 13 females; M:F = 1.5:1; median age 54 years, range 31–82) were deemed eligible to receive oxaliplatin–fluoropyrimidine chemotherapy in addition to SSA; 14 received XELOX and 18 FOLFOX. After a median follow-up of 26 mo., each patient had completed at least two cycles of chemotherapy. The ORR was 25%, with a median DoR of 21.3 mo. The DCR was 87.5%. Notably, 28.1% of patients experienced tumor shrinkage sufficient for radical surgery on residual tumor lesions, encompassing both primary tumors and metastases; (4) Conclusions: Upfront treatment with the combination of oxaliplatin–fluoropyrimidine and SSA demonstrated effectiveness and safety. This approach may be considered to facilitate conversion surgery in eligible patients.

## Simple Summary

Gastroenteropancreatic neuroendocrine tumors (GEP-NETs) are often diagnosed at an advanced stage. While somatostatin analogs (SSAs) are the first-line treatment for well-differentiated somatostatin receptor-positive (SSTR+) NETs, they may be insufficient for patients with G2/G3 tumors and high tumor burden. This retrospective study evaluated the efficacy of combining oxaliplatin–fluoropyrimidine chemotherapy with SSA in 32 patients with metastatic G2/G3 GEP-NETs. After a median follow-up of 26 months, the objective response rate (ORR) was 25%, with disease control in 87.5% of cases and tumor shrinkage allowing surgery in 28.1% of patients. Median progression-free survival (PFS) and overall survival (OS) were not reached. The treatment was well tolerated, with mostly mild adverse events. This combination therapy was effective and safe, potentially enabling curative surgery in eligible patients.

## 1. Introduction

Neuroendocrine tumors (NETs) are a wide group of neoplasms arising from the diffuse neuroendocrine cell system [1], with an incidence ranging between 1 and 5 per 100,000 individuals (M: 2/100,000, F: 2.4/100,000) [2], steadily rising lately. NETs are frequently diagnosed at advanced stage and the gastro-entero-pancreatic tract is the primary site (Gastro-Entero-Pancreatic neuroendocrine Tumors, GEP-NETs), especially the small-intestine (SI-NETs) and pancreas NETs (Pan-NETs) [3,4]. NETs can be classified according to the new WHO classification in four categories based on tumor grade, cancer cell morphology, proliferation index (Ki-67 index), and mitotic count as well as the presence of necrosis: well-differentiated NETs G1, G2, and G3 and poorly differentiated neuroendocrine carcinomas (NECs), representing 10–20% of all neuroendocrine neoplasms [5]. This classification along with primary site and TNM stage are the most relevant prognostic factors [6]. Once obtained a definite diagnosis and staging, choosing the most appropriate treatment is not always easy. Usually, for patients with unresectable or advanced disease, systemic treatment with palliative intent is the standard of care, with an estimated median overall survival (OS) between 2 and 5 years [1]. In this setting, Somatostatin Analogues (SSAs) are the preferred option as first line therapy [7,8]. Other treatment options include the mechanistic targeting of rapamycin (m-TOR) inhibitors such as everolimus [9,10], tyrosine kinase inhibitors (TKI) like sunitinib [11,12], and chemotherapy [13,14,15,16,17,18,19,20], but these are usually reserved to progressive disease. Recently, Peptide Receptor Radionuclide Therapy (PRRT) with Lutetium-177 (177Lu) DOTATATE has garnered significant attention as a valuable treatment option both in naïve and pre-treated metastatic/inoperable NETs that showed homogenous SSTR expression by SSA-positive Positron Emission Tomography/Computed Tomography (PET/CT) or single photon emission computed tomography (SPECT) imaging [21,22,23]. Yet, despite the availability of various treatments, until now there is little evidence concerning the best first line strategy and no direct comparison is available. In patients with advanced disease, particularly in newly diagnosed patients with distant metastases and high tumor burden (e.g., unresectable liver metastases or multiple metastatic sites involved), SSA treatment might not be enough. There is often a short time to progression, usually reported on first tumor evaluation imaging, suggesting a low efficacy of SSA therapy alone. In these cases, combining the SSA with another treatment might be an effective strategy to reduce the burden of disease, delaying time to radiological progression and clinical worsening, eventually impacting survival. Most of the data on chemotherapy combination efficacy are derived from small retrospective series or single-arm phase II studies that mainly enroll Pan-NET patients [24]. The aim of our monocentric study was to evaluate the efficacy of the combination of oxaliplatin–fluoropyrimidine chemotherapy in association with a cold somatostatin analog therapy as up-front strategy in patients with newly diagnosed metastatic well-differentiated GEP-NET.

## 2. Materials and Methods

We conducted a retrospective observational study on patients of 18 years or older with histologically confirmed newly diagnosed metastatic well-differentiated GEP-NET G2/G3 referred to the European Neuroendocrine Tumor Society (ENETS)-certified Centers of Excellence (CoE) of our Institution, Fondazione Policlinico Universitario “Agostino Gemelli”—IRCCS in Rome. Data were collected from patients’ medical records. Previously untreated patients with metastatic GEP-NET G2/G3 who had undergone SSA treatment combined with an oxaliplatin–fluoropyrimidine chemotherapy were considered. Demographic and clinicopathological information was collected. Each patient at baseline underwent a complete morphological imaging work-up with contrast-enhanced magnetic resonance (RMI) and/or a computed tomography (CT-scan) of the thoracic district and the upper and lower abdomen at baseline at three-month intervals for response assessment. Disease response was defined according to the Response Evaluation Criteria In Solid Tumors version 1.1 (RECIST 1.1) criteria [25]. We also collect results of disease functional characterization by PET/CT with 68-Gallium-DOTATOC (68-Ga) and 18F-Fluorodeoxyglucose (18F-FDG). Patients with functional NETs were excluded from our study as well as patients with a diagnosed genetic syndrome such as multiple endocrine neoplasia 1 (MEN-1) or Von Hippel–Lindau (VHL) disease. Mixed neuroendocrine/non-endocrine neoplasms (Mi-NEN) cases were also excluded. All patients underwent cold somatostatin analog therapy with lanreotide 120 mg every 28 days. Only those who started the cytotoxic treatment within three months of starting treatment with SSA without evidence of disease progression were considered eligible for analysis. Oxaliplatin–fluoropyrimidine chemotherapy was administered according to one of the following schemes: modified FOLFOX-6 (oxaliplatin 85 mg/m^2^ ev, on day 1 q.14; 5-fluorouracil 400 mg/m^2^ ev, as a bolus, on day 1 q.14; 5-fluorouracil 2400 mg/m^2^ ev, as continuous infusion, on days 1 and 2 1 q.14; folinic acid 200 mg/m^2^ ev, on day 1 q.14) or XELOX (oxaliplatin 130 mg/m^2^ ev, on day 1 q.21; capecitabine 2000 mg/m^2^ oral, on days 1–14 q.21). Treatment was continued until progression, unacceptable toxicity, or withdrawal of consent. Dose reduction or delay followed clinical practice. All patients enrolled received at least two cycles of oxaliplatin–fluoropyrimidine chemotherapy treatment. The primary endpoint was the overall response rate (ORR). Progression-free survival (PFS) was considered a co-primary endpoint. The secondary endpoints were the disease control rate (DCR), the duration of response (DoR), and overall survival (OS). For the evaluation of treatment-related toxicity, the Common Terminology Criteria for Adverse Events (CTCAE) version 5.0 was used. An exploratory analysis was performed to evaluate the survival impact of demographic and clinicopathological factors as age, sex, primary tumor location, metastatic site distribution, grading, KI 67 index, expression of SSTR2A by immunohistochemistry (IHC) according to Volante score [26].

Statistical analyses were performed using SPSS^®^ software (SPSS for Windows Version 29.0; SPSS Inc, Chicago, IL, USA). Baseline characteristics were expressed as counts and percentages if categorical, means with standard deviation if continuous and normal or median with interquartile range if continuous not normal. Normality was checked using the Shapiro–Wilk test. Difference between categorical variables was tested using the Chi-squared test while differences between continuous variables were assessed using the Student T test or the Mann–Whitney test. Survival was described using the Kaplan–Meier method with mean estimates and reported with hazard ration and their 95% Confidence Intervals (95%CI). Survival outcomes were compared using the log-rank test. Cox-regression analysis was used to assess, both singularly and collectively, the weight of clinically relevant covariates on survival outcomes. Statistical significance was declared at two-sided *p* < 0.05.

## 3. Results

### 3.1. Demographic and Baseline Characteristics of Patients

From March 2017 to October 2023, 32 patients with newly diagnosed metastatic well-differentiated G2/G3 GEP-NET coming to our Institution were considered eligible by our Multidisciplinary Tumor Board dedicated to NETs to an oxaliplatin–fluoropyrimidine treatment in addition to SSA. Nineteen were males and thirteen females (M:F = 1.5:1). The patients’ ages at baseline spanned from 31 to 82 years, with a median age of 54 years (interquartile range 20.5 years, 44.1–64.6 years). The baseline clinicopathological characteristics of the patient population are shown in Table 1.

### 3.2. Study Outcomes

**Figure 1:**
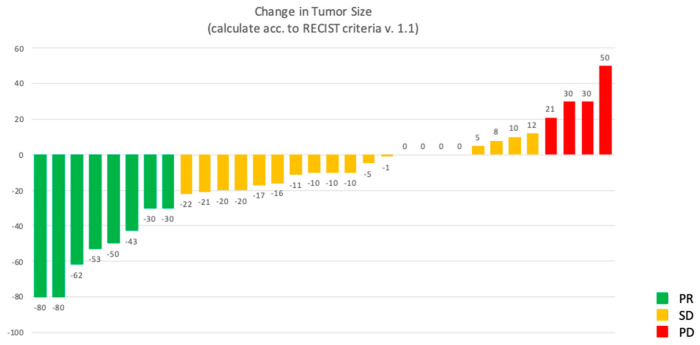
Waterfall plot of responses.

**Figure 2:**
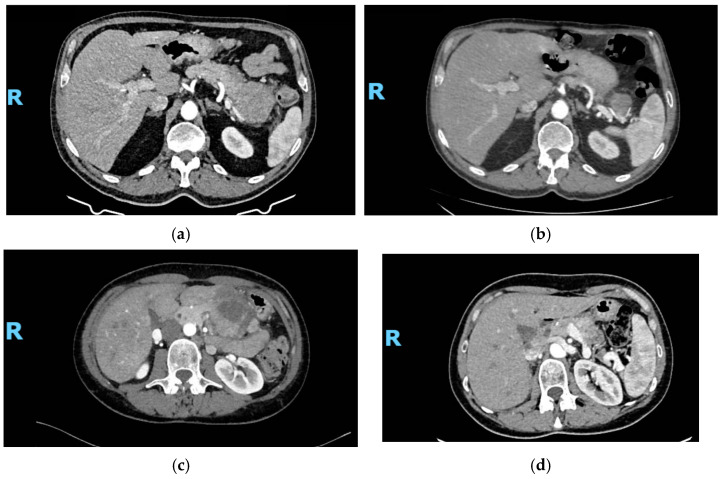
Best response in two patients treated with FOLFOX + SSA: (**a**–**c**) CT-scans at baseline; (**b**–**d**) CT-scans after VI cycles. R: right.

At the data cut-off analysis in October 2023, after a median follow-up of 45.9 months (mo.) each patient completed at least two cycles of oxaliplatin–fluoropyrimidine chemotherapy treatment. Eleven patients were treated with XELOX and seventeen with FOLFOX. For all patients, SSA treatment was prescribed within three months from day one of the first cycle of chemotherapy. The median time under oxaliplatin–fluoropyrimidine plus SSA combination therapy was 11.4 mo. Of the 32 patients enrolled, at data cut-off, 12 patients (37.5%) progressed or died, while 20 (62.5%) were still in follow-up. Up to 22 patients experienced tumor shrinkage (68.7%), with an overall response rate (ORR) according to RECIST v. 1.1 of 25% with 8 patients reporting a partial response (PR) as best response on treatment, while 20 patients (62.5%) had disease stability (SD). Figure 1 shows the waterfall plot of responses. The median duration of response (DoR) was 21.3 mo. Disease control rate (DCR) was 87.5%. Four patients (12.5%) were primary refractors to study treatment combination and had PD as best response at the first tumor evaluation. Despite no complete response being seen according to RECIST v. 1.1, in nine patients (28.1% of the overall population) tumor shrinkage was enough to allow a radical surgery on residual tumor lesions, including both primitive tumor and metastases, even in cases of high burden of disease at baseline. Figure 2 shows an example of significant tumor shrinkage in two pancreatic NET patients referred to radical surgery after treatment. The median PFS in the overall population was not reached (NR) due to paucity of events. The estimated mean PFS of the entire population at data cut off was 51.8 mo. (95%CI 38.3–65.3 mo.) (Supplementary Materials, Figure S1). Similarly, median overall survival (OS) has not yet been achieved (NR), due to the low number of events. The estimated mean OS was 62.2 mo. (95%CI 51.1–73.2 mo.) (Supplementary Materials, Figure S2).

**Figure 3:**
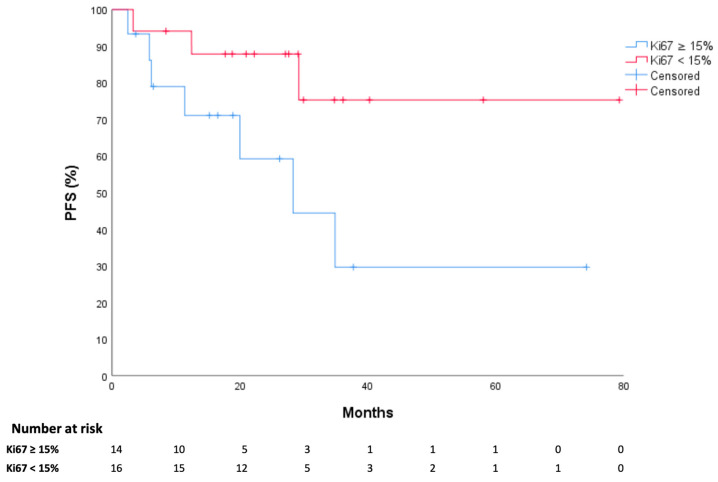
Progression-Free Survival (PFS) according to Ki67 Index (cut-off 15%).

Demographic factors such as age and sex did not influence patients’ survival outcomes. Regarding the disease characteristics, there was a numeric difference in PFS between patients with GEP-NETs G2 and G3, despite it not being statistically significant (*p* = 0.28) probably due to the small sample size and the overrepresentation of G2 cases. We therefore stratified the patients according to Ki67 lower or higher than 15%, showing a statistically significant difference. Patients with Ki67 < 15% had a longer PFS than patients with Ki67 ≥ 15%: 64.3 mo. (95%CI 49.0–79.6 mo.) versus 35.5 mo. (95%CI 18.4–52.6 mo.) (*p* = 0.047) (Figure 3). Stratifying the patients according to the somatostatin receptor IHC score (<2+ versus ≥2+), no differences were found in terms of PFS. Any difference was seen based on primary tumor location or metastatic sites.

**Figure 4:**
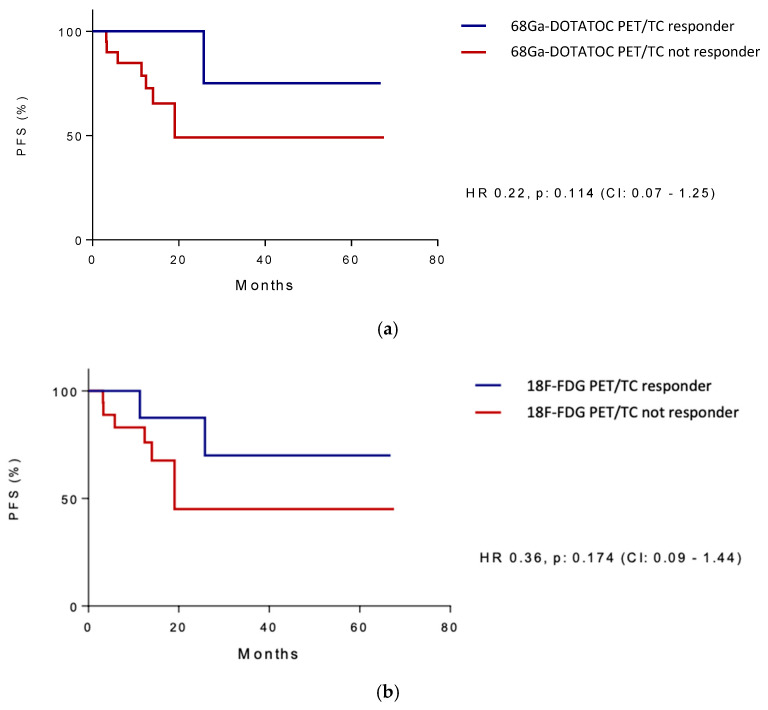
Progression-Free Survival (PFS) according to metabolic response to 68Ga-DOTATOC PET/CT (**a**) and 18F-FDG PET/CT (**b**).

Twenty-eight patients enrolled in this analysis underwent a complete PET/CT functional evaluation by performing a dual-tracer evaluation both with 68-Ga-DOTATOC and 18F-FDG. All these patients had an SSA-positive PET/CT disease, while twenty patients were found to be positive and eight negative to 18F-FDG PET/CT, respectively. Of note, for ten patients (35.7%), 18-FDG and 68Ga-DOTATOC PET/CT were concordant, with both tracers showing a pathologically improved uptake in the majority of tumors, while more than a half (64.3%) had discordant results. 18F-FDG PET/CT positivity was evaluated as a stratifying prognostic factor. When analyzing patients’ PFS based on the uptake of PET/CT with 18F-FDG, a different trend emerged between the two groups: as predicted, in patients with positive 18F-FDG PET/CT, the PFS was inferior, reaching 36.0 mo. (95%CI 18.9–53.2 mo.) versus 56.8 mo. (95%CI 38.1–75.6 mo.) in subjects with negative 18F-FDG PET/CT with an HR of 0.26 (95%CI 0.08–1.57). Nevertheless, these results were not statistically significant (*p* = 0.182). We further evaluate the PFS according to the metabolic response evaluated by PET/CT with both tracers used. Patients achieving a metabolic partial or complete response had a better prognosis irrespective of the response evaluated by CT or MRI according to RECIST v. 1.1. (Figure 4).

Regarding safety, Table 2 lists and classifies the main adverse events that occurred according to CTCAE v. 5.5. Of all patients, 75% experienced at least a mild (G1, G2) adverse event, while the incidence of G3 events was around 15%, leading to chemotherapy dose delay and/or reduction in 5 patients. The most frequent adverse events were anemia, oxaliplatin-related neurotoxicity, and fatigue. No patient was discontinued due to drug related toxicity.

## 4. Discussion

GEP-NETs pose a challenge for medical oncology. Their unpredictable aggressiveness and clinical outcomes put caused clinicians to re-think their therapeutic strategy, particularly for high-grade G2/G3 GEP-NET. Despite SSAs showing efficacy in advanced well-differentiated NETs, their antitumor effect is predominantly cytostatic: their use was associated with a slight benefit in time to progression, with a greater benefit in indolent G1 and G2 GEP-NETs with Ki67 <10%, while response rate was low (<5%) [7,8]. So far, when early tumor shrinkage is an urgent need for a patient, because of critical site metastatic involvement, invalidating symptoms or high disease burden, a more effective cytotoxic therapy should be proposed. In heavily symptomatic disease, high tumor burden and/or metastases located in potentially critical sites, a treatment approach that cam ensure early tumor shrinkage and warrant a durable response might be taken into account. In this retrospective analysis, we presented our knowledge of oxaliplatin–fluoropyrimidine schemes use in a population of patients affected by newly diagnosed unresectable advanced/metastatic G2/G3 GEP-NETs with a more aggressive clinical or radiological presentation. This patient population represents the ideal setting for an up-front use of SSAs combined with another more effective treatment, without waiting for clinical progression. Although no definitive conclusions may be drawn from our study due to its retrospective nature and the relatively small sample size, interesting observations could be made. In the overall study population, we obtained an ORR of 25%, more than double compared to historical data in the literature regarding chemotherapy efficacy (11.5% ranging from 5.8% to 17.2%) [13,14,15,16,17,18,19,20], similar to everolimus plus SSA (26.8%in G1-G2 patients) [27], but different to PRRT-induced response rates in the NETTER-2 trial (43% in somatostatin receptor-positive tumors) [23]. In 87.5% of the subjects treated with XELOX or FOLFOX, a durable disease control was obtained with a median DoR of 21.3 months after achieving a RECIST v. 1.1 response. Despite there being no complete response according to RECIST v. 1.1, in nine patients (28.1% of the overall population) tumor shrinkage was enough to permit a radical surgery on residual tumor sites, including both primitive tumor and metastases, even in case of high-burden disease at baseline. According to international guidelines, surgery should be attempted in the presence of resectable or potentially resectable metastases, especially liver metastases, whenever possible, even in case of a high disease burden to palliate symptoms. Previous data revealed that a curative resection of GEP-NETs with liver metastases is associated with a 5-year OS rate around 85% [28]. Yet, a real curative resection (R0, R1) is difficult to achieve in advanced/metastatic disease and could delay the start date for systemic therapy. Our series suggests a role for a pre-operative systemic treatment, allowing a conversion surgery with radical intent in a not neglectable percentage of patients even with high-burden disease. This supports the suggestion that pursuing surgery is meaningful for NET patient outcomes, even in the metastatic setting, as demonstrated by previous relevant series focusing on the impact of primary tumor resection [29,30]. Median PFS and OS was not reached due to short follow-up and a low rate of events. Although not mature, PFS and OS data appear promising. In our study, we stratified the patients according to the histological and metabolic characteristics of the tumor. A significant trend towards survival benefit emerged based on Ki67 values above or below 15%, in line with the literature. No difference emerged based on an SST2A score of ≤ 2 or 3. In this regard, it should be remembered that scores of 2 and 3 represent a high expression of SSTR2A, which agrees with response to therapy in Volante’s experience [26]. Our results suggest not to adopt this criterion in patient selection for proposing or excluding cytotoxic treatment. Another explored association was between functional images by PET/CT and outcomes. Increased disease uptake by 18F-FDG PET/CT evaluation appears to be a poor prognostic factor for metastatic GEPNET [31]. For enrolled patients who underwent both a 68-Ga-DOTATOC and 18F-FDG PET/CT, a correlation between PFS and 18F-FDG PET/CT uptake emerged: in patients with positive 18F-FDG PET/CT, the PFS was inferior in negative subjects, although not statistically significant. The addiction of cytotoxic treatment combination does not seem capable of modifying the more unfavorable prognosis of patients with 18F-FDG-positive disease, though it might be a selection criterion for an up-front chemotherapy approach. The oxaliplatin–fluoropyrimidine and SSA combination treatment was well tolerated, toxicity was in line with expectations, and no toxicity-related deaths were reported. All reported adverse events were mild or moderate, and improved with correct management; the most frequent were oxaliplatin-related neurotoxicity, anemia, and fatigue. No patients discontinued chemotherapy due to severe toxicity. The major limit of our study is the methodology of retrospective analyses itself, including selection bias and potential confounders. The limited number of patients enrolled and the low rate of events also affected statistical analyses. Therefore, prospective studies with a larger sample size and a longer follow-up are needed. The lack of a standard control arm is another key point for a future study design.

## 5. Conclusions

An upfront treatment with an oxaliplatin–fluoropyrimidine and SSA combination is effective and safe in newly diagnosed patients with metastatic well-differentiated G2/G3 GEP-NETs, providing prolonged progression-free survival. This strategy should be offered as a valid option in the treatment of advanced/metastatic GEP-NETs in selected patients with high-risk features (Ki67 ≥ 15%; 18-FDG PET/CT scan positive; high tumor burden; clinical impairment). Long-term disease control and even conversion surgery are major goals that could also permit chemotherapy breaks and de-escalation, alleviating treatment-related toxicity and improving patients’ quality of life.

## Figures and Tables

**Table 1:** Patients’ clinicopathological characteristics.

Characteristics	Patients Numbers (%)
Primitive Tumor Location	
Pancreas	19 (59.3)
Small Intestine	5 (15.6)
Other	8 (25)
- Stomach	2 (6.2)
- Liver	1 (3.1)
- Unknown	5 (15.6)
Grading acc. WHO	
G2	26 (81.2)
G3	6 (18.8)
Metastatic site distribution	
Liver	23 (71.9)
Lymph nodes	19 (59.3)
Bone	13 (40.6)
Lung	7 (21.9)
Peritoneum	5 (15.6)
SSTR2A by IHC acc. Volante Score	
0	2 (6.2)
1	0 (0)
2	3 (9.4)
3	27 (84.4)

PS: performance status; ECOG: Eastern Cooperative Oncology Group; WHO: World Health Organization; PET/CT: Positron Emission Tomography/Computed Tomography; 68-Ga: 68-Gallium-DOTATOC; 18F-FDG: 18F-Fluorodeoxyglucose; SSTR2A: somatostatin receptor type 2A; IHC: immunohistochemistry.

**Table 2:** Study treatment-related adverse events according to CTCAE v. 5.0.

CTCAE Term	Incidence (%)			
	G1	G2	G3	G4
**Paresthesia**	21.8	15.6	3.1	0
**Neutropenia**	9.3	0	3.1	0
**Febrile Neutropenia**	-	-	-	0
**Platelet Count Decrease**	3.1	9.3	6.2	0
**Anemia**	25	0	0	0
**Nausea/Vomiting**	18.7	9.3	0	0
**Oral Mucositis**	6.2	0	0	0
**Diarrhea**	12.5	12.5	3.1	0
**Fatigue**	21.8	15.6	0	0

## Data Availability

The authors confirm that the data supporting the findings of this study are available within the article. Any other information is available from the corresponding author, MGM, upon reasonable request.

## Supplementary Materials

The following supporting information can be downloaded at: https://www.mdpi.com/article/10.3390/cancers17091561/s1, Figure S1: Kaplan Meyer Survival curve for Progression-Free Survival, Figure S2: Kaplan Meyer Survival curve for Overall Survival.
