# Supplementary material for: Upfront Oxaliplatin–Fluoropyrimidine Chemotherapy and Somatostatin Analogues in Advanced Well-Differentiated Gastro-Entero-Pancreatic Neuroendocrine Tumors
Source: Cancers (Basel). 2025 May 3;17(9):1561. doi: 10.3390/cancers17091561 (PMC12071586; doi:10.3390/cancers17091561)

Supplementary

Figure S1. Progression-Free Survival (PFS) for the study population.

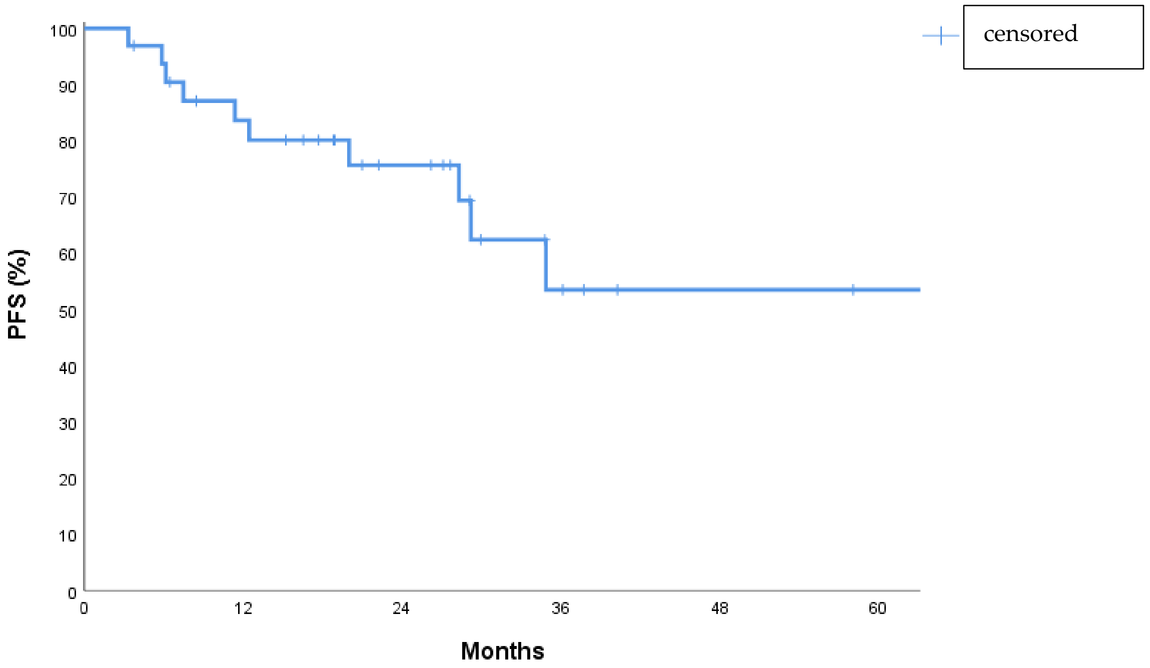

Figure S2. Overall Survival (OS) for the study population.

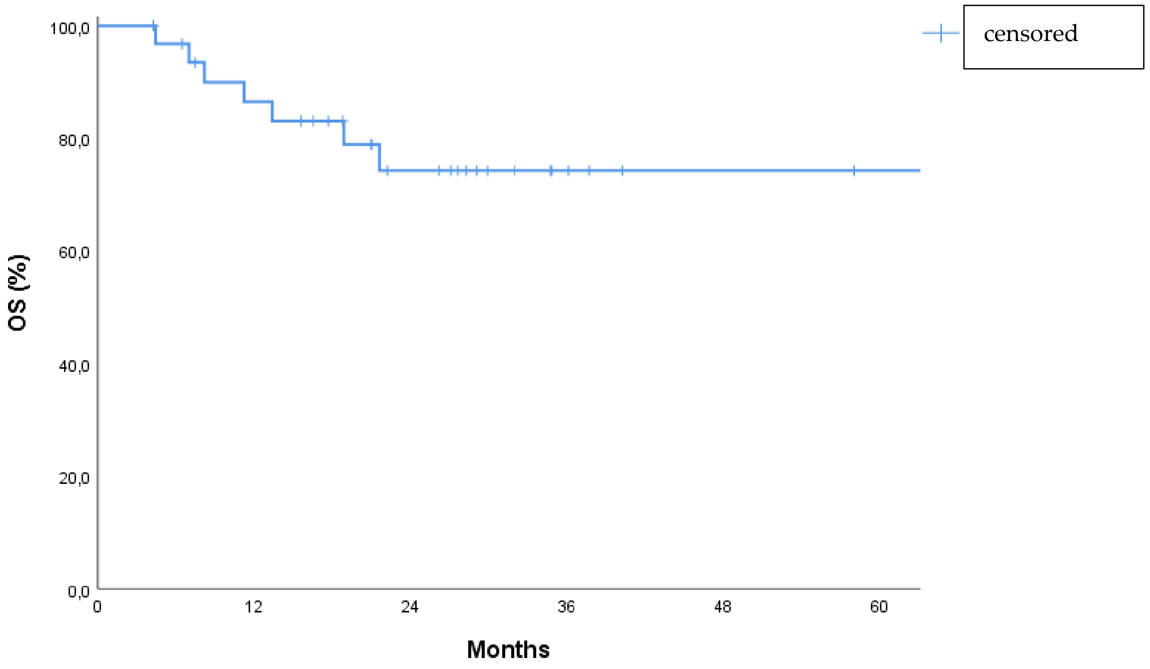

Supplement: Supplementary file 1 [file cancers-17-01561-s001.zip › cancers-3592694-supplementary.pdf]
